# Generation and characterization of ultrathin free-flowing liquid sheets

**DOI:** 10.1038/s41467-018-03696-w

**Published:** 2018-04-10

**Authors:** Jake D. Koralek, Jongjin B. Kim, Petr Brůža, Chandra B. Curry, Zhijiang Chen, Hans A. Bechtel, Amy A. Cordones, Philipp Sperling, Sven Toleikis, Jan F. Kern, Stefan P. Moeller, Siegfried H. Glenzer, Daniel P. DePonte

**Affiliations:** 1 SLAC National Accelerator Laboratory, Menlo Park, CA 94720 USA; 2 ELI Beamlines, Institute of Physics of the Czech Academy of Sciences, Na Slovance 2, Prague, 18221 Czech Republic; 3 Thayer School of Engineering, Dartmouth College, 14 Engineering Dr, Hanover, NH 03755 USA; 4 Department of Electrical and Computer Engineering, University of Alberta, Edmonton, AB T6G 1H9 Canada; 5 Advanced Light Source, Lawrence Berkeley National Laboratory, Berkeley, CA 94720 USA; 6 European X-Ray Free-Electron Laser Facility GmbH, Schenefeld, 22869 Germany; 7 Deutsches Elektronen-Synchrotron, DESY, Notkestraße 85, Hamburg, D-22607 Germany

## Abstract

The physics and chemistry of liquid solutions play a central role in science, and our understanding of life on Earth. Unfortunately, key tools for interrogating aqueous systems, such as infrared and soft X-ray spectroscopy, cannot readily be applied because of strong absorption in water. Here we use gas-dynamic forces to generate free-flowing, sub-micron, liquid sheets which are two orders of magnitude thinner than anything previously reported. Optical, infrared, and X-ray spectroscopies are used to characterize the sheets, which are found to be tunable in thickness from over 1 μm  down to less than 20 nm, which corresponds to fewer than 100 water molecules thick. At this thickness, aqueous sheets can readily transmit photons across the spectrum, leading to potentially transformative applications in infrared, X-ray, electron spectroscopies and beyond. The ultrathin sheets are stable for days in vacuum, and we demonstrate their use at free-electron laser and synchrotron light sources.

## Abstract (web summary)

X-ray spectroscopy is a tool used for the investigation of aqueous solutions but the strong absorption of water means that very thin liquid sheets are needed for accurate analysis. Here the authors produce free-flowing liquid sheets 2 orders of magnitude thinner than sheets obtained with existing techniques.

## Introduction

Much of the chemistry critical to life on Earth occurs in a liquid environment, and achieving a better understanding of these processes requires that measurements be made in the liquid state. Indeed, much of what is known about biochemical reactions has been learned from photon spectroscopy on species in aqueous solutions. However, there remain regions of the electromagnetic spectrum for which spectroscopy of liquids is impractical due to strong absorption. The infrared (IR) and soft X-ray regions of the spectrum, e.g., are critical for understanding basic physics, biology, and chemistry, but are difficult to apply to liquids due to the need for sub-micron thick samples. Even the electronic structure of water itself remains elusive due to the difficulty in obtaining barrier-free thin samples.^1,2^ Sub-micron cylindrical liquid jets have been used for spectroscopic applications^3^, but photon scattering and refraction due to the cylindrical liquid boundary, as well as reduced signal from mismatch between jet diameter and focus size, are often problematic. Flat liquid sheets can be made wide enough to eliminate these problems^4–7^, but have not previously been available with sub-micron thickness or sample flow rates below 1 mL min^−1^.

Here we demonstrate generation of free-flowing liquid sheets, with thickness tunable down to 10’s of nm, using a microfluidic nozzle operating in the 150 μL min^−1^ flow range. At this thickness, these sheets can be used for IR, X-ray and even electron spectroscopy, and will also be advantageous for high-resolution time-resolved techniques. The ultrathin sheets can operate stably in vacuum for days, and we demonstrate their application to soft X-ray and IR spectroscopy.

## Results

### Gas-dynamic sheet generation

**Fig. 1:**
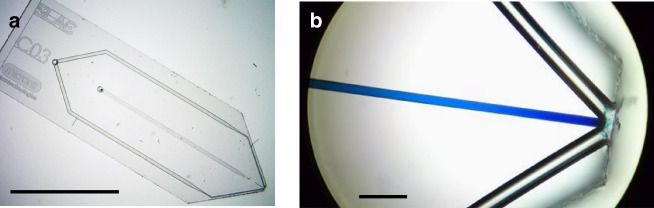
Microfluidic gas-dynamic nozzle. The microfluidic device for generating free-flowing liquid sheets is shown in **a**. The gas and liquid ports are on the underside of the chip on the left. Microfluidic channels for gas (outer), and liquid (central) can be traced to the output of the nozzle on the right side of the chip. The chip dimensions are 6×19 mm, and the scale bar is 6 mm. A close-up of the nozzle output is shown in **b**, where a blue dye has been introduced into the liquid channel. The scale bar in **b** is 100 μm

The device used for generating ultrathin liquid sheets is a gas-dynamic nozzle^3,8,9^ consisting of three microfluidic channels in a borosilicate chip. The devices used for this study were fabricated with photolithography (Methods section), but other materials and fabrication techniques, such as 3D printing, could be used to fabricate a similar device. Figure 1a shows a microscope image of the microfluidic chip housing the sheet nozzle. As shown in the figure, the gas and liquid inputs are on the left hand side of the chip, and the microfluidic channels can be traced to the exit of the nozzle on the right side of the chip. Figure 1b shows a higher magnification image of the nozzle exit geometry, where two 50 μm diameter channels intersect a central 20 μm channel (containing blue liquid) at angles of ±40° before exiting the chip.

**Fig. 2:**
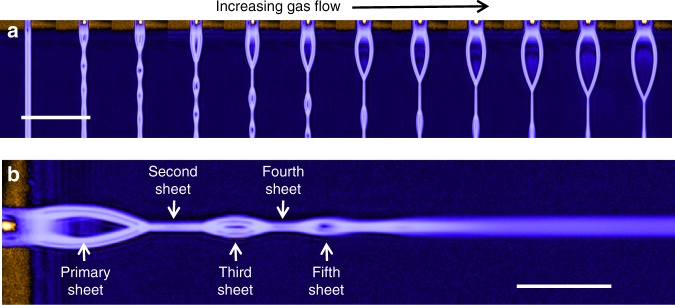
Ultrathin liquid sheet generation. A series of images depicting the formation of a liquid sheet is shown in **a**. In these images the nozzle output is on the top, the liquid is flowing downward, and the gas flow increases as we move to the right of the figure. The cylindrical liquid jet on the far left evolves into a thin sheet as gas flow is increased. The scale bar in **a** is 1 mm. A more detailed view of the alternating orthogonal sheet structure is shown in **b**, where the nozzle is on the left, and the liquid is flowing to the right. The scale bar in **b** is 500 μm

The generation of liquid sheets is depicted in Fig. 2, where panel a shows a series of images where the nozzle exit is on the top, and blue liquid is flowing downward. The liquid flow rate was kept constant, but the gas flow rate was increased incrementally, starting at zero on the left hand side, and reaching 100 SCCM on the right. The cylindrical liquid jet on the left side of Fig. 2a is gradually spread into a sheet as the speed of the colliding gas jets is increased with increasing gas flow. The physics underlying this sheet formation is similar to that underlying the formation of a sheet by two colliding cylindrical liquid jets (we note that the device described here can also be used to form a sheet by the collision of two cylindrical jets, but discussion of that will be reserved for future publication).^10–14^ As in the case of colliding liquid jets, the colliding gas jets here impart radial momentum to the liquid, causing it to spread into a thin sheet, bounded by a thicker fluid rim. The sheet becomes progressively thinner moving outward along the streamlines and, in the case of vacuum operation, must also rapidly decrease in temperature due to evaporative cooling^15^. Downstream from the gas–liquid interaction point, surface tension eventually overcomes the radial momentum, causing the thick rims of the sheet to re-converge, which then in turn produces a smaller sheet in the orthogonal orientation. This process repeats, resulting in a series of alternating, orthogonal sheets, as shown in Fig. 2b. The sheet closest to the nozzle, the primary sheet, gets progressively wider and thinner as gas flow is increased, allowing in situ control of the sheet dimensions. Such control is not possible in sheets formed with colliding liquid jets or converging nozzles, where the thickness is independent of liquid flow velocity^13^. The use of a focusing gas allows us to reduce the liquid flow rate by an order of magnitude relative to colliding liquid jets, resulting in much thinner sheets. The gas also protects the liquid from freezing near the nozzle in vacuum environments.^16^

### Optical spectroscopy

**Fig. 3:**
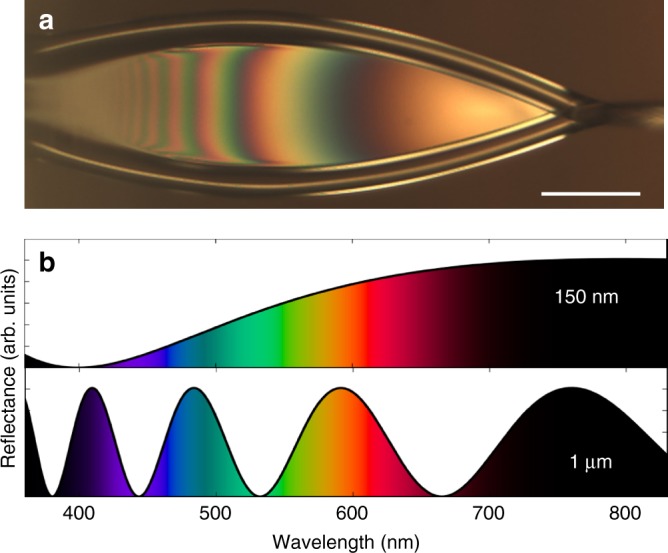
Spectral reflectance of water. An optical reflection image of an ultrathin liquid sheet is shown in **a**, highlighting the thin-film interference fringes. The calculated spectral reflectance of water for films of 150 nm and 1 μm thickness are shown in **b**. The scale bar in **a** is 50 μm

**Fig. 4:**
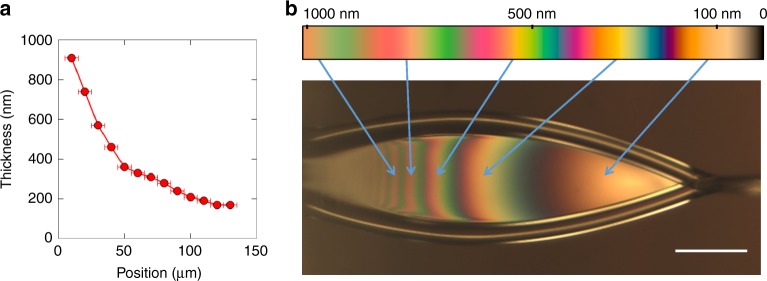
Optical characterization of liquid sheets. The thickness of a liquid sheet is measured by spectral reflectance, as described in the text, and plotted in **a** as a function of position along the sheet, starting at the far left arrow in **b**. The error bars in **a** represent the 10 μm sampling size of the measurement. The color bar above the image in **b** is a simulation of the observed reflected color for water films, as a function of thickness, as described in the text. Arrows indicate the corresponding fringes on the liquid sheet, in agreement with the spectral reflectance measurements of **a**. The scale bar in **a** is 50 μm

A fair amount of information can be inferred from analysis of optical images of the liquid sheets. Figure 3a shows a reflection image of a water sheet running in air, at a flow rate of 250 μL min^−1^, taken with a color CCD camera through ×10 long working distance objective and fiber-coupled tungsten light source (Ocean Optics HL-2000-FHSA at 2960 K). Thin-film interference fringes can clearly be seen from the sheet, demonstrating the surface quality and integrity of the sheet. These thin-film interference fringes can also be used to estimate the thickness of the sheet. In the thin-film interference regime, a film of liquid has a spectral reflectance curve that depends strongly on thickness,^17^ as illustrated in Fig. 3b where the simulated spectral reflectance of water is plotted for 1 μm and 150 nm films. This strong variation allows us to determine the sheet thickness by measuring the reflectance spectrum using a fiber-coupled spectrometer (Thorlabs CCS200) and then comparing to the calculated spectra as a function of thickness. The illumination spot, and spectrometer in-coupling, are scanned along the length of the sheet, and the reflectance spectrum is used to determine the thickness at each point. The result of this procedure is plotted in Fig. 4a, which shows the observed thickness as a function of position along the center of the sheet. Here the uncertainty largely stems from the finite sampling spot size of the spectrometer, which in this case is estimated to be 10 μm diameter.

The rapidly varying reflectance spectra along the length of the sheet leads to interference fringes of distinct perceived colors, which can be captured by a color CCD camera as can be seen in Fig. 4b. Above the CCD image is a color bar where we plot the simulated color variation with thickness for a water film, calculated using the measured index of refraction of water^18^ and assuming a 3000 K blackbody illuminating spectrum. Although we did not account for the camera sensitivity or viewing medium, the sheet thickness estimated this way agrees well with the spectral measurements of Fig. 4a. We conclude that the sheet in Fig. 4b goes from about 1 μm near the nozzle to about 100 nm close to the re-convergence point. We note that this is not the thinnest sheet possible, but rather one in the right thickness range to best illustrate the thin-film interference.

**Fig. 5:**
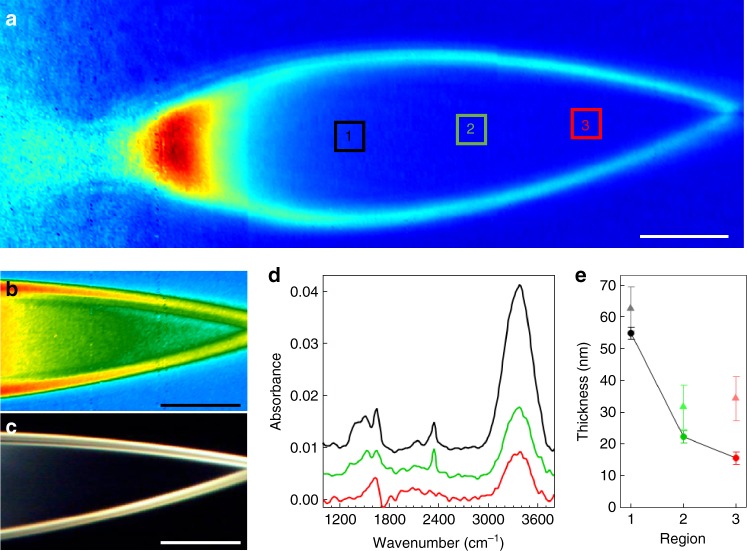
Infrared spectromicroscopy of ultrathin liquid water sheets. **a** A false-color image of the integrated IR transmission (3100–3650 cm^−1^) through a water sheet with 150 µL min^−1^ flow rate, compiled from three separate tiled images with no additional processing. The color scale for the image in **b** is the spectral weight in the O–H stretch mode, highlighting the amount of liquid water in the sheet. The corresponding optical reflection image is shown in **c**, demonstrating total lack of reflection for the thinnest parts of the sheet. **d** Infrared absorption spectra of the water sheet in the three regions highlighted by the black, green, and red boxes in **a**. The spectra for regions 1 and 2 are offset for clarity. **e** The calculated sheet thickness in each of the three regions indicated in **a**, using the measured absorbance values of the O–H stretch at 3404 cm^−1^ (dark circles, connected by black lines for clarity) and the H_2_O bending mode at 1643.5 cm^−1^ (light triangles). The error bars in **e** represent an estimate of the uncertainty of the absorption measurement based on the signal-to-noise ratio of the peaks of interest. In this case, the error bars are calculated by measuring the baseline noise (peak-to-peak) on either side of the peak of interest and scaling the error for the calculated thickness by standard error propagation methods. The scale bars in all panels are 50 μm

Thin-film interference is a very useful tool for optimizing and assessing sheet thickness, flatness, and stability in situ. However, it becomes increasingly less reliable as sheets become much thinner than the wavelength of the illuminating source, a few hundred nm for visible light. For the thinnest sheets we generated, the tip of the sheet fades from yellow to black, as essentially no reflection is detected from the thinnest portion of the sheet. This can be seen by comparing the sheet in Fig. 4b, where the thinnest portion of the sheet reflects yellow, to Fig. 5c, where the lower half of the sheet reflects no measurable visible light. In order to measure the thickness of water sheets below 100 nm, we used IR transmission spectromicroscopy as described in the following section.

### Infrared spectromicroscopy

Infrared spectroscopy in liquids has historically been hindered by the fact that water must be no more than a few microns thick in order to readily transmit IR radiation. Liquid spectroscopy is typically accomplished using IR-transmissive cells, however, the cell walls must then be taken into account when analyzing the data. The free-flowing ultrathin liquid sheets presented here clearly represent an opportunity for IR spectroscopy. In this section we demonstrate the utility of ultrathin sheets for IR spectroscopy, and use IR transmission to determine the thickness of our thinnest sheets, which are too thin to be determined by visible spectral reflectance.

We imaged the liquid sheets at beamline 2.4 at the Advanced Light Source (ALS), using an Agilent Cary 620 FTIR microscope fitted with a ×15 all-reflective objective (NA = 0.62)  and a 128×128 HgCdTe pixel-array detector. This system simultaneously captures Fourier transform infrared (FTIR) spectra on each detector pixel, giving us a spatial map of the IR spectra for the entire sheet. For these measurements, a globar source and KBr beamsplitter were employed. The FTIR spectrometer and microscope were purged with nitrogen, except for the space between the objectives where the sheet jet was run in air and directed into a ~1 cm diameter vacuum hose pumped with a diaphragm pump. Helium was used as the accelerating gas, adding no background as it is not IR active. Figure 5 displays data acquired with this system on a sheet jet running at 150 μL min^−1^. Figure 5a shows a false-color image of the total integrated IR transmission over the range 3100–3650 cm^−1^, assembled from three tiled images with no additional processing. We note that the sheet in Fig. 5a is slightly asymmetric due to an imperfection in the chip manufacturing process that has since been corrected. Figure 5b shows only the far end of a similar sheet in a different false-color scale representing the integrated spectral weight in the O–H stretch mode from roughly 3100 to 3550 cm^−1^. It effectively shows the amount of liquid water in the sheet, and corroborates the optical reflection data indicating an intact sheet that gets gradually thinner moving away from the nozzle towards the re-convergence point. We also note that this spectral weight map largely excludes any potential contribution from the O–H stretch mode of gaseous water, which would appear outside this range at higher energy relative to the observed liquid peak. At this flow rate, the sheet is too thin for thin-film interference in the visible spectrum and optical reflection can no longer accurately indicate the thickness for the lower half of the sheet, where essentially no reflection is detected, as shown in Fig. 5c. We can still observe variation of the IR transmission in the region, and can measure a transmission of over 98% at the O–H stretch mode absorption peak.

Figure 5d shows baseline-corrected absorbance spectra, spatially averaged over three distinct regions of interest, highlighted by colored boxes in Fig. 5a. We use the Beer–Lambert equation to calculate the sheet thickness from the measured absorbance,

1 $${\mathrm{Absorbance}} = \log \left( {\frac{{I_0}}{I}} \right) = \varepsilon lc$$

where $\varepsilon$ is the molar absorptivity, *c* is the concentration of water (55.5 M), and *l* is the sheet thickness. The thickness obtained for the three regions is plotted in Fig. 5e, for both the O–H stretch mode (circles) and H_2_O bend mode (triangles). For the O–H stretch mode in region 3 we measure an absorbance of 0.0087 ± 0.0009 at 3404 cm^−1^. Using the literature value of $\varepsilon$ = 00.61 ± 0.02 M^−1^cm^−1^(ref.^19^), we calculate a sheet thickness of just 16 ± 2 nm. We note that the maximum absorbance of 0.0097 ± 0.0009 for the corresponding spectrum in Fig. 5d occurs at 3375 cm^−1^, which is red-shifted from the literature value^19^. If we use the maximum absorbance value with the literature molar absorptivity at 3404 cm^−1^, the calculated sheet thickness is increased only slightly to 17 ± 2 nm. Following a similar procedure for the H_2_O bending mode at 1643.5 cm^−1^ we measure an absorbance of 0.0041 ± 0.0008, which corresponds to a thickness of 34 ± 7 nm using $\varepsilon$ = 21.65 ± 0.07 M^−1^cm^−1^(ref.^19^). The source of this discrepancy in thickness as measured between the two modes is not known at this point. However, thicker sheets (>200 nm) show much better agreement between the two modes, (Methods section) and nearly quantitative agreement with the optical measurements in Fig. 4. Because the thinner sheets are near the detection limit of the setup, background subtraction errors, scattering effects, and other artifacts have a larger impact on the analysis. The bending mode is particularly affected at lower signal levels because of its weaker molar absorptivity, and the fact that the frequency range overlaps with water vapor absorption, making it more susceptible to errors in the thin sheet limit. Indeed, the spectra in Fig. 5d show clear indications of spectral contamination at frequencies just below the H_2_O bending region. We therefore place higher emphasis on the stretch measurements, which indicate a sheet thickness of 16 ± 2 nm in region 3.

### Demonstration of soft X-ray measurements

Ultrathin liquid sheets have potential for use in a wide variety of X-ray measurements, most notably in the soft X-ray regime, where sub-micron penetration depths have severely limited measurements on liquids. The ultrathin sheets presented here not only allow for transmission of soft X-rays, but also offer a superior geometry compared with sub-micron round jets. Since the typical X-ray focus at a synchrotron or free-electron laser (FEL) is larger than 1 µm, a round jet thin enough for transmission will necessarily capture only a small fraction of the beam. This not only reduces the signal of interest, but also causes scattering from the edge of the jet, which can be a significant source of background. This scattering from the edge of round jets is particularly problematic in imaging experiments where it is often the largest signal on the detector. The sheets described here were recently used at the FLASH facility in Hamburg and the SXR beamline at the Linac Coherent Light Source (LCLS) at the SLAC National Accelerator Laboratory. The main scientific findings of those experiments will be presented elsewhere, but a description of the sheet performance is given below.

In the case of the experiment performed at FLASH, a liquid water sheet was used to study the electronic and thermal properties of isochorically heated water. The 225 eV photon pulses from FLASH were used to excite the water sheet, and subsequent dynamics were monitored by transmission and reflection of visible light pulses from a femstosecond optical parametric amplifier (OPA). A flowing water sheet was necessary in order to present a flat water-surface for reflection and transmission measurements, which would not be possible with a cylindrical jet. The experiment was performed with the sheet running with a water flow rate of 250 μL s^−1^ and a Helium flow rate of 100 SCCM, in a vacuum chamber at a pressure typically around 10^−3^ Torr. Under these conditions the sheet was able to operate continuously for up to 48 h while being illuminated by the OPA (8 μJ, 90 fs, 1 mm collimated) and excited by the FEL (20 μJ, 100 fs, 25 μm focus) at 10 Hz.

**Fig. 6:**
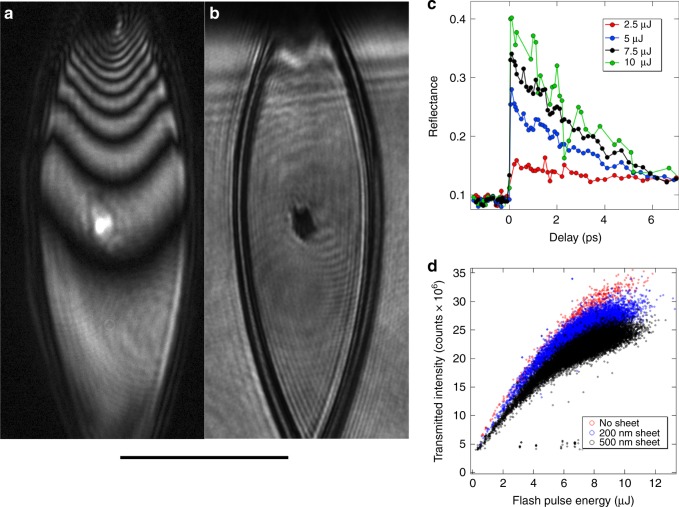
Soft X-ray measurements of liquid water sheets. Measurements from the FLASH free-electron laser are shown as described in the text. Shown are optical reflection (**a**) and transmission (**b**) images taken in situ. The entire sheet was illuminated by an optical parametric amplifier (500 nm), which was used as a probe of the dynamics induced by the soft X-ray pulses. The X-ray spot can be seen as a bright spot in reflection and a dark spot in transmission, immediately following the X-ray pump. **c** The reflectivity of the X-ray pumped region of the water sheet, as a function of time delay between the X-ray pump and optical probe, for a series of different pump fluences. **d** The intensity of soft X-rays transmitted through the water sheet, as a function of FLASH pulse energy, for different sheet thicknesses. The scale bar is 100 μm

Figure 6 shows optical reflection and transmission images taken during the experiment. Thin-film interference from the OPA illumination can be seen in the reflection image (Figure 6a), and was used to monitor the stability and thickness of the sheet. In this case, interpretation of the thin-film interference fringes is simplified by illumination with a monochromatic source, giving accurate measurement of sheet thickness in situ. The FEL pulse excites free charge carriers near the critical density of the 500 nm probe (the critical density is 4.4 × 10^21^ per cc, and the estimated density in our experiment is 2–4 × 10^21^ per cc). The high electron density leads to transient metal-like behavior, resulting in increased reflection and simultaneously reduced transmission. Hence, the X-ray spot can be seen as a bright spot in the reflection image  (Figure 6a) and a dark spot in the transmission image (Figure 6b), both taken immediately following excitation. The dynamics are extracted by monitoring the change in reflectivity (within the bright spot) as a function of time delay between the X-ray pump and optical probe as plotted in Fig. 6c for several different pump intensities. We use the measured reflection and transmission data to find the complex index of refraction of the FEL-heated water. Assuming the charge carriers (conduction electrons) behavior in the excited water can be described by a Drude model, we estimate the charge carrier density of 2–4 × 10^21^ per cc, which indicates ~10% ionization of water (3.34 × 10^22^ water molecules per cc). This number is significantly higher than the direct photo-ionized electron density by direct FEL light absorption of a single electron (the absorbed FEL photon density is ~1–2 × 10^20^ per cc), and thus indicating the importance of secondary processes that will need to be taken into account in future models. We also monitored the intensity of FLASH photons transmitted through the water sheet, as a function of incident pulse energy, which is plotted in Fig. 6d.

**Fig. 7:**
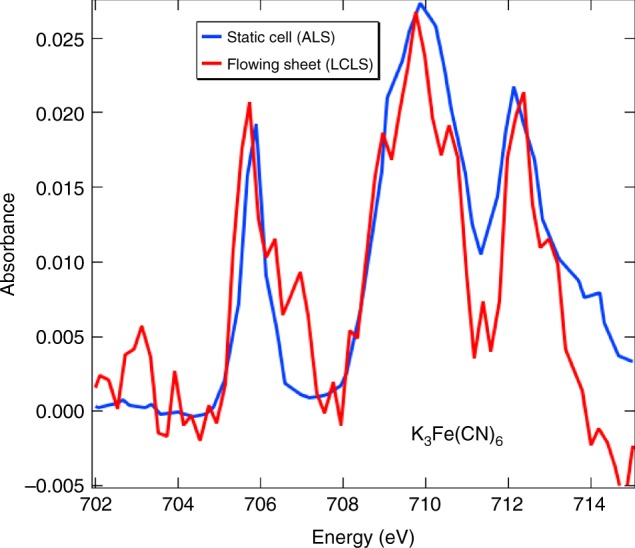
Soft X-ray transmission spectroscopy of aqueous sheets. Soft X-ray transmission spectra for 100 mM Fe(CN)_6_^(3−)^ measured at the ALS in a static cell (blue), and at the LCLS in a flowing ultrathin liquid sheet (red) are shown. The ALS data was scaled down to compare to the absorbance of the much shorter liquid path in the LCLS data

For the experiment carried out at LCLS, a 100 mM K_3_Fe(CN)_6_ aqueous liquid sheet, flowing at 300 μL min^−1^, was used to measure the Fe L_3_-edge X-ray absorption spectrum, in transmission geometry, at the SXR beamline. Synchrotron absorption data were also collected, for the same sample, at the ALS, beamline 6.0.2, in Berkeley, for comparison using a static liquid cell. For the synchrotron measurement, the sample thickness was 2 μm, and the cell walls were 100 nm Si_3_N_4_. Due to the fluid being static at the ALS, the X-ray fluence and flux had to be kept very low to avoid damage; a photon flux around 6 × 10^7^ photons s^−1^ in a 50×75 μm focus was used. The LCLS data was acquired under very different conditions, as the ultimate goal of the experiment was to measure short timescale, transient response, to an optical excitation. This requires extremely high intensity FEL pulses to get the needed signal with the intended temporal resolution; pulse energy of 1 μJ, 50 fs duration, 120 Hz repetition rate and a spot size of 25×54 μm was used. This corresponds to 10^12^ photons s^−1^ incident on the sheet, which is five orders of magnitude higher than was used at the ALS. A static cell could not be used with such a high intensity source, necessitating a free-flowing liquid sheet. A cylindrical jet could also not be used because such a jet thin enough for X-ray transmission would necessarily be much smaller than the X-ray focus, and the non-uniform thickness probed in the transmission geometry would lead to spectral distortion. Furthermore, a cylindrical jet also acts as a lens focusing the optical pump, resulting in non-uniform excitation of the sample. The sheet used was approximately 600 nm thick and was operated in vacuum at a pressure of 10^−4^ Torr. Figure 7 shows a comparison of the absorbance of K_3_Fe(CN)_6_, as measured at the ALS (blue) and at the LCLS (red). ALS data was acquired for 25 s per data point (0.5 s per data point per scan, average of 50 scans), and the LCLS data was acquired for 34 s per data point. In both cases the photon energy was scanned with a monochromater. Despite the differences between the ALS and LCLS experimental arrangements, the data are in good agreement, demonstrating the applicability of ultrathin liquid sheets to high-field soft X-ray FEL measurements.

## Discussion

We have demonstrated free-flowing liquid sheets below 20 nm thick, generated by gas-dynamic forces from a microfluidic device. The use of gas-dynamic forces has many advantages over existing sheet jet systems based on hydrodynamic forces, such as reduced liquid flow rate and sheet thickness, in situ tunability of sheet dimensions, and long-term stability in vacuum. Such thin liquid sheets are potentially transformative for IR, soft X-ray, and electron spectroscopies, which all suffer from strong absorption in liquid water. We successfully deployed the liquid sheet jet at LCLS, ALS and FLASH, and presented optical, IR and X-ray data from these experiments, demonstrating the practical viability of ultrathin liquid sheets in spectroscopy and imaging experiments.

## Methods

### Microfluidic chip fabrication

The microfluidic chips were made by Micronit Microtechnologies BV using standard hard lithography methods. Two 0.4 mm thick borosilicate wafers were etched with symmetric patterns, bonded, and diced. The etching process creates channels that extend 2.5 μm beyond the mask features, resulting in oval shaped channels that are 5 μm wider than their depth. Several prototypes were tested with gas channels converging at ±20°, ±30°, and ±40° relative to the central channel, and various distances, *D*, from the point of channel convergence to chip exit. The largest angle of 40° and shortest available *D* of 25 μm qualitatively appeared to make widest sheets and so were chosen for this study. Gas channels of 50 μm and 40 μm were tested, and no qualitative difference in functionality was observed. Data for Figs. 1, 5, and 7 was taken using 50 μm gas channels, while the remaining data was taken using 40 μm channels.

### Sheet jet operation

For the measurements described here, de-ionized water (or other liquid sample) was supplied to the central channel of the microfluidic chip using an HPLC pump connected to a pulsation dampener. Helium gas flow was controlled with either a manual regulator or a mass flow controller. We find that stable liquid sheets can be formed for flow rates above about 150 μL min^−1^. The gas flow required to form a sheet increases as liquid flow rate is increased, as do the physical dimensions of the sheet. Stable sheet operation is typically achieved using gas flow rates around 100 SCCM at a liquid flow rate of 250 μL min^−1^.

### Direct comparison of IR and optical spectroscopy

**Fig. 8:**
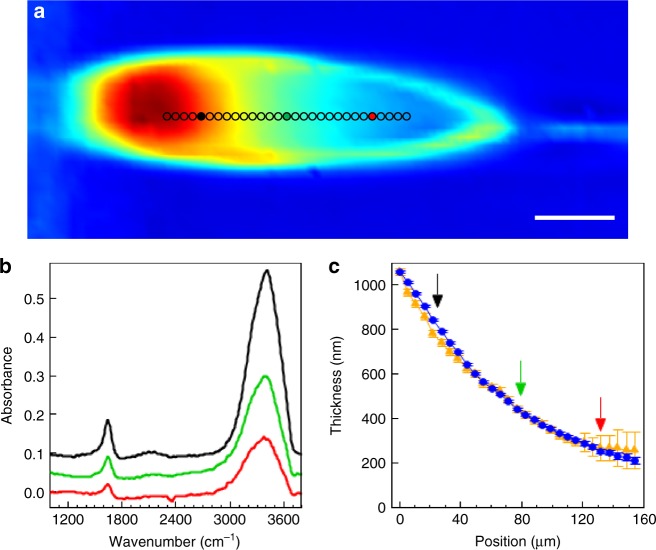
Infrared spectromicroscopy of liquid water sheets. **a** A false-color image of the integrated IR transmission (3100–3650 cm^−1^) through a water sheet with 250 µL min^−1^ flow rate, under similar conditions as Fig. 4. The scale bar is 50 µm.** b** The infrared spectra of the water sheet at three points along the sheet, as indicated by the black, green, and red colored circles in **a**. The black and green spectra are offset for clarity. **c** The calculated sheet thickness along the central region of the water sheet, as indicated by the markers in **a**, using values of the O–H stretch mode at 3404 cm^−1^ (blue circles) and the H_2_O bending mode at 1643.5 cm^−1^ (gold triangles). The black, green, and red arrows indicate the positions of the respective colored markers in **a**, as well as the colored spectra in **b**. The error bars in **c** represent an estimate of the uncertainty of the absorption measurement based on the signal-to-noise ratio of the peaks of interest. In this case, the error bars are calculated by measuring the baseline noise (peak-to-peak) on either side of the peak of interest and scaling the error for the calculated thickness by standard error propagation methods

The optical reflectance data of Figs. 3 and 4 were measured on a sheet optimized to best show thin-film interference, running at 250 μL min^−1^. The IR data of Fig. 5 was taken on a sheet optimized for minimum thickness, running at 150 μL min^−1^. In order to allow a direct comparison of sheets running under identical conditions, we provide Fig. 8, which shows IR spectromicroscopy data for a water sheet running at 250 μL min^−1^, similar conditions to the sheet in Figs. 3 and 4. Figure 8a shows an IR transmission image, with the color scale representing the integrated IR transmission (3100–3650 cm^−1^). The absorbance at the O–H stretch mode at 3404 cm^−1^, and the H_2_O bending mode at 1643.5 cm^−1^, were used to estimate the sheet thickness via the Beer–Lambert equation, as in the main text, which is plotted as a function of position for both modes in Fig. 8c. Figure 8b shows three representative IR transmission spectra, taken from the positions indicated by the appropriately colored arrows in Fig. 8c, and filled circles in Fig. 8a. Under these operating conditions, the thickness profile of the sheet, as measured by IR transmission (Fig. 8c) and optical reflection (Fig. 4a), agree qualitatively, and even quantitatively very well. With the IR measurements, we obtain full spectra for each pixel of the image, and so can measure over a larger range than is possible with the optical measurements, which are averaged over a 10 μm spot. All IR spectra shown were baseline corrected by subtracting a polynomial fit to regions outside the H_2_O stretching and bending modes. We note that the images in fig. 5 were aquired in high-mag mode with an effective pixel size of 1.1 μm, while the image in fig. 8 was aquired in low-mag mode with an effective pixel size of 5.5 μm.

### Data availability

The data that support the findings of this study are available from the corresponding author upon reasonable request.
