# Author Correction: Generation and characterization of ultrathin free-flowing liquid sheets

**DOI:** 10.1038/s41467-018-05365-4

**Published:** 2018-07-17

**Authors:** Jake D. Koralek, Jongjin B. Kim, Petr Brůža, Chandra B. Curry, Zhijiang Chen, Hans A. Bechtel, Amy A. Cordones, Philipp Sperling, Sven Toleikis, Jan F. Kern, Stefan P. Moeller, Siegfried H. Glenzer, Daniel P. DePonte

**Affiliations:** 1 SLAC National Accelerator Laboratory, Menlo Park, CA 94720 USA; 2 ELI Beamlines, Institute of Physics of the Czech Academy of Sciences, Na Slovance 2, Prague 18221, Czech Republic; 3 Thayer School of Engineering, Dartmouth College, 14 Engineering Dr, Hanover, NH 03755 USA; 4 Department of Electrical and Computer Engineering, University of Alberta, Edmonton, AB T6G 1H9 Canada; 5 Advanced Light Source, Lawrence Berkeley National Laboratory, Berkeley, CA 94720 USA; 6 European X-Ray Free-Electron Laser Facility GmbH, Schenefeld 22869, Germany; 7 Deutsches Elektronen-Synchrotron, DESY, Notkestraße 85, Hamburg D-22607, Germany

Correction to: *Nature Communications*; 10.1038/s41467-018-03696-w; published online 10 April 2018.

The original version of this Article omitted the following from the Acknowledgements:

‘P.B. was funded by the ELI Extreme Light Infrastructure Phase 2 (CZ.02.1.01/0.0/0.0/15008/0000162) from the European Regional Development Fund and the EUCALL project funded from the EU Horizon 2020 research and innovation programme under grant agreement No 654220,’ which replaces the previous ‘P.B. was funded by the ELI Extreme Light Infrastructure Phase 2 (CZ.02.1.01/0.0/0.0/15008/0000162) from the European Regional Development Fund.’

This has been corrected in both the PDF and HTML versions of the Article.

