# Author Correction: Generation and characterization of ultrathin free-flowing liquid sheets

**DOI:** 10.1038/s41467-019-09457-7

**Published:** 2019-04-03

**Authors:** Jake D. Koralek, Jongjin B. Kim, Petr Brůža, Chandra B. Curry, Zhijiang Chen, Hans A. Bechtel, Amy A. Cordones, Philipp Sperling, Sven Toleikis, Jan F. Kern, Stefan P. Moeller, Siegfried H. Glenzer, Daniel P. DePonte

**Affiliations:** 1 SLAC National Accelerator Laboratory, Menlo Park, CA 94720 USA; 2 ELI Beamlines, Institute of Physics of the Czech Academy of Sciences, Na Slovance 2, Prague, 18221 Czech Republic; 3 Thayer School of Engineering, Dartmouth College, 14 Engineering Dr, Hanover, NH 03755 USA; 4 Department of Electrical and Computer Engineering, University of Alberta, Edmonton, AB T6G 1H9 Canada; 5 Advanced Light Source, Lawrence Berkeley National Laboratory, Berkeley, CA 94720 USA; 6 European X-Ray Free-Electron Laser Facility GmbH, Schenefeld, 22869 Germany; 7 Deutsches Elektronen-Synchrotron, DESY, Notkestraße 85, Hamburg, D-22607 Germany

Correction to: *Nature Communications* 10.1038/s41467-018-03696-w; published online 10 April 2018

The original version of this Article contained an error in Equation (1), which incorrectly read “${\mathrm{Absorbance}} = \log \left( {\frac{{I_0}}{I}} \right) = \varepsilon lh$”.

The correct version reads “${\mathrm{Absorbance}} = \log \left( {\frac{{I_0}}{I}} \right) = \varepsilon lc$”. This has been corrected in both the PDF and HTML versions of the Article.

